# Developing microsatellite duplex PCR reactions for sterlet (*Acipenser ruthenus*) and their application in parentage identification

**DOI:** 10.1038/s41598-022-16194-3

**Published:** 2022-07-14

**Authors:** Jun Wang, Zhiwei Sun, Linlang Jiang, Yacheng Hu

**Affiliations:** 1 State Key Laboratory of Eco-Hydraulics in Northwest Arid Region of China, Xi’an University of Technology, Xi’an, 710048 China; 2 Chinese Sturgeon Research Institute, China Three Gorges Corporation, Yichang, 443100 Hubei China

**Keywords:** Genetic markers, Inbreeding

## Abstract

The sterlet (*Acipenser ruthenus*) is one of the 27 sturgeon species and is well-known for its wide distribution and small body size in comparison to other sturgeons. For assessing the population genetics and parentage identification of sterlet, ten microsatellites developed for Chinese sturgeon and cross-amplified in sterlet were tested by 40 individuals of sterlet. The ten microsatellites were developed using transcriptome sequencing of Chinese sturgeon. The expected heterozygosity (*H*_*E*_), observed heterozygosity (*H*_*O*_), Shannon-Weiner diversity indices (*H′*) and polymorphic information content (*PIC*) of the 10 microsatellites ranged from 0.466 to 0.751, from 0.438 to 0.938, from 0.66 to 1.51 and from 0.368 to 0.716, respectively. Combined exclusion probability based on the genotype of pair parent known (CE-PP), one parent known (CE-2P), and no parent known (CE-1P) of the 10 microsatellites were 99.99%, 99.96%, and 99.49%, respectively. These result showed that the 10 microsatellites should be helpful for assessing the population genetics and parentage identification of sterlet.

Sturgeon is famous for its caviar. Due to the influence of human activities, the number of all sturgeon is decreasing. The sterlet (*Acipenser ruthenus*, Linnaeus, 1758) is one of the 27 sturgeon species and is well-known for its wide distribution and small body size in comparison to other sturgeons^1^. Genetic breeding of farmed sterlet is an effective means to meet the demand for human consumption of caviar and food, as well as for conservation of wild sturgeon resources. The sexual maturation period of sterlet is usually 2–3 years and 3–4 years for males and females, respectively. Therefore, sterlet is an ideal model to study the sturgeon due to earliest maturation^2^. The aquaculture development of sterlet has two major bottlenecks, which is the larval rearing and the broodstock reproduction^3^. Due to many years of artificial breeding, inbreeding has become an urgent problem in the breeding of small sturgeons.

Microsatellite refer to simple sequence repeats (SSRs) or short tandem repeats (STRs) and are considered as tracts of DNA motifs, which is ranging from one to 10 nucleotides in length, and the DNA motifs is repeating of 5–50 times^4^. Microsatellite play an important role on studying the structure and the genetic diversity of populations for supportive stocking programs. Microsatellite can be used to evaluate animals breeding and develop pedigree animal populations, supporting genetic improvement^5^. The microsatellite DNA has been widely used for genetic analysis of aquatic animals, such as *Acipenser sinensis*^6^, *Exopalaemon carinicauda*^7^, *O. niloticus*^8^, *Acipenser dabryanus*^9^, *Acipenser schrenckii*^10^, and *Siniperca scherzeri*^11^. Moreover, duplex PCR panels for microsatellite loci co-amplification can reduce time and costs in genetic analysis of aquaculture animals. Microsatellite for genetic studies of the sterlet have been developed^12^. In that study, six species-specific microsatellites for starlet were developed to examined in 67 sterlet individuals (20 farmed and 47 wild-caught), and the result suggested the wild sterlet population had indications of inbreeding. However, no study has been reports about parentage identification for sterlet. The application of microsatellites using in parentage verification have produced highly effective and accurate results^13^. Therefore, there is a need for developing microsatellites that are suitable for accurate parentage testing in sterlet. Hence, in the present study, we aimed to develop microsatellites that are should be helpful for accurate parentage testing in sterlet.

## Methods

### Ethics statement

The present study was approved by the Animal Administration and Ethics Committee of Xi’an University of Technology. All experiments were carried out in accordance with relevant guidelines and regulations. The study was carried out in compliance with the ARRIVE guidelines.

### Sample collection

Chinese sturgeon (*Acipenser sinensis*) using in transcriptome assembly was collected in the Chinese Sturgeon Research Institute, (Yichang, China). The 16 individuals of sterlet were randomly selected from the corresponding cultures in a farm in Xi’an, China. These individuals were used for validating polymorphic microsatellite markers. In addition, A full-sib families of sterlet was obtained by artificial insemination in the farm. 22 progenies and their parents were collected for DNA isolation. A total of 40 individuals were used in this study.

### DNA isolation

The fin of each individuals was taken and preserved in alcohol. Genomic DNA was extracted from fin of all samples of sterlet. The standard proteinase-K digestion and phenol/chloroform method was used for DNA isolation. The fin of sterlet was put in the tube with 10 µl proteinase-K for 50 min at 56 °C. Subsequently, 600 µl of extraction buffer (0.5 M Tris pH 8.0, 0.5 M EDTA, 5 M NaCl, 10% SDS) was added to this tube, and incubated for 10 min at 95 °C. Then, 500 µl phenol was put in the tube. The mixture was violented shock for 10 s and centrifuged for 15 min at 12000 g at 4 °C. 200 µl supernatant of mixture was transferred to a fresh tube. 250 µl chloroform was put this tube, and the mixture was centrifuged for 15 min at 12,000 g at 4 °C. The supernatant of mixture was transferred to another a fresh tube. 500 µl isopropanol was put this tube, and the mixture was centrifuged for 15 min at 12,000 g at 4 °C. The supernatant of mixture was discarded and 1 ml 75% ethanol was put in this tube to precipitate the pellet. And the mixture was centrifuged for 15 min at 12,000 g at 4 °C, and the supernatant of mixture was discarded. At last, the pellet was dissolved using 200 µl sterile water. 1% agarose gel was used to test DNA integrity.

### Transcriptome assembly

The fin of Chinese sturgeon was used to extract RNA using Trizol (Invitrogen, USA) following the manufacturer’s instructions. 1% agarose gel was used to test RNA integrity. Then, the RNA of Chinese sturgeon was used to construct cDNA libraries by using Illumina truSeq stranded mRNA sample preparation protocol. The Illumina HiSeq™ 4000 with 100 bp paired-end sequencing was used to sequence the cDNA libraries with 100 or 2 × 90 bp paired-end sequencing. SOAPnuke (v1.5.6)^14^ was used to filter the raw reads of libraries according to three criteria: (i) reads with ≥ 20% bases Q ≤ 20 were removed, (ii) reads with a high proportion of unknown sequences were removed (> 5%), (iii) reads with contained adaptors (adaptors with ≤ 20% mismatches were allowed) were discarded^15^. The Trinity program (version: release-2013-08-14) was used to assemble the clean reads. The TGICL^16^ was used to cluster the transcripts. The redundant sequences were eliminated and the non-redundant sequences of > 200 bp were retained.

### Identification of microsatellites

The Microsatellite Identification tool^17^ was used to select sequences that is constraining perfect repeat motifs of 4 bp from the libraries data. And the Microsatellite Identification tool was set for search criteria for identification of at least 6 repeat units. The Primer Premier 5.0 software^18^ was used to design microsatellite primers from all the selected sequences. The primer pairs flanking each microsatellite was identified with a annealing temperature of an optimum at 60 °C, PCR products with expected lengths between 100 and 400 bp.

### Marker selection

The DNA of 16 randomly individuals of sterlet were used to screen for polymorphic microsatellites and to optimize amplification conditions. PCR amplification was performed in 25 μl volumes containing 0.25 μM each primer, 1.5 mM MgCl_2_, 0.25 U of Taq polymerase (Takara, China), 0.25 μM PCR buffer (Takara, China), 0.25 μM dNTPs, about 50–100 ng of template DNA, and ultra-pure water. PCR amplification was conducted under the following conditions: an initial step at 94 °C for 3 min, followed by 35 cycles of denaturing 30 s at 94ºC, 60 °C for 30 s, and 72 °C for 30 s, with a final prolonging at 72 °C for 10 min. The PCR products were resolved with 10% polyacrylamide gel electrophoresis (PAGE). The pBR322 DNA/Mspl marker (Takara) was used as a molecular size standard.

### Establishing duplex PCR panels for sterlet and their application in parentage identification

According to the result of marker selection of sterlet, duplex PCR reactions was chosen. The primer dimers and potential hairpin structures must be avoided in choosing of duplex PCR reactions. If primer dimers and potential hairpin structures appear in PCR reaction products, this combination of primers is filtered out. Five duplex PCR reactions were assembled, each containing two microsatellites. The duplex PCR reactions were performed at a total volume of 25 μl, containing 0.25 μM each primer, 1.5 mM MgCl_2_, 0.5 U of Taq polymerase (Takara, China), 0.5 μM PCR buffer (Takara, China), 0.5 μM dNTPs, about 80–100 ng of template DNA, and ultra-pure water. The duplex PCR reactions were performed under the following profile: an initial step at 94 °C for 3 min, followed by 35 cycles of denaturing 30 s at 94 °C, 60 °C for 30 s, and 72 °C for 30 s, with a final prolonging at 72ºC for 10 min. The PCR products were resolved with 10% polyacrylamide gel electrophoresis (PAGE). The pBR322 DNA/Mspl marker (Takara) was used as a molecular size standard. The selected microsatellites for duplex PCR reactions were used for parentage identification of the full-sib families of sterlet.

### Genetic analysis

The ATetra1.2 software^19^ was used to calculate observed heterozygosity (*H*_*O*_), the mean expected heterozygosity (*H*_*E*_), and Shannon-Weiner diversity indices (*H′*). The polymorphic information content (*PIC*) was calculated using the formula $PIC=1-\sum_{i=1}^{n}{P}_{i}^{2}-\sum_{i=1}^{n-1}{\sum }_{j=i+1}^{n}2{P}_{i}^{2}{P}_{j}^{2}$, *P*_*i*_ and *P*_*j*_ are the frequencies of I and J allele in each microsatellite loci. The Gene Marker software was used to calculate the size of allele. Microsoft Office Excel 2007 was used to process the genotypic data. Based on the allele phenotypes, a dendrogram of ten random individuals of sterlet was constructed using MEGA software^20^. The FaMoz software^21^ was used to calculate the exclusion and combined exclusion probability based on the allele frequencies. During the analysis the genetic data of the experiment, the dropout and false allele should be avoid.

## Results

**Figure 1:**
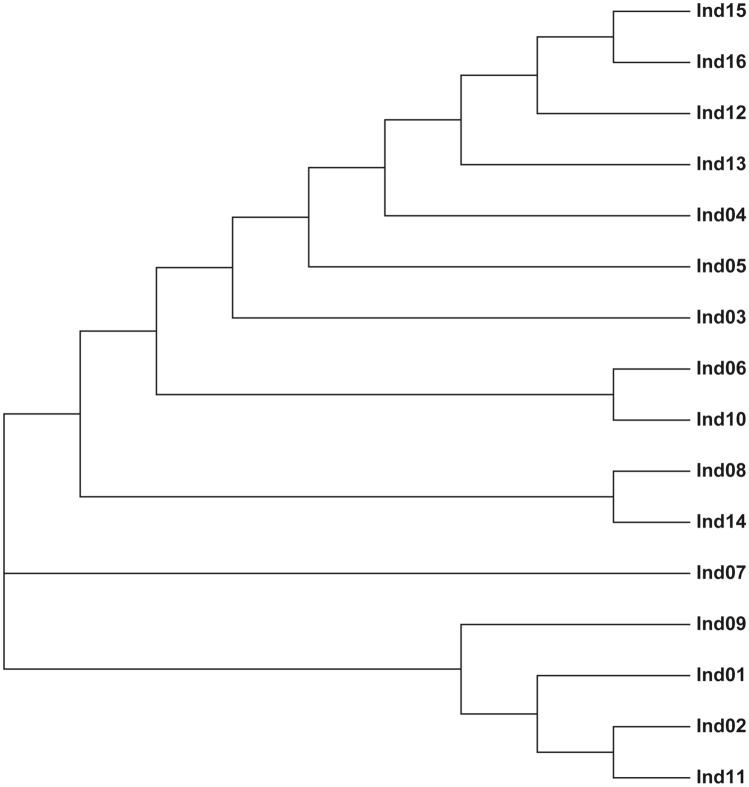
A dendrogram of 16 random individuals of sterlet was constructed using MEGA software.

**Figure 2:**
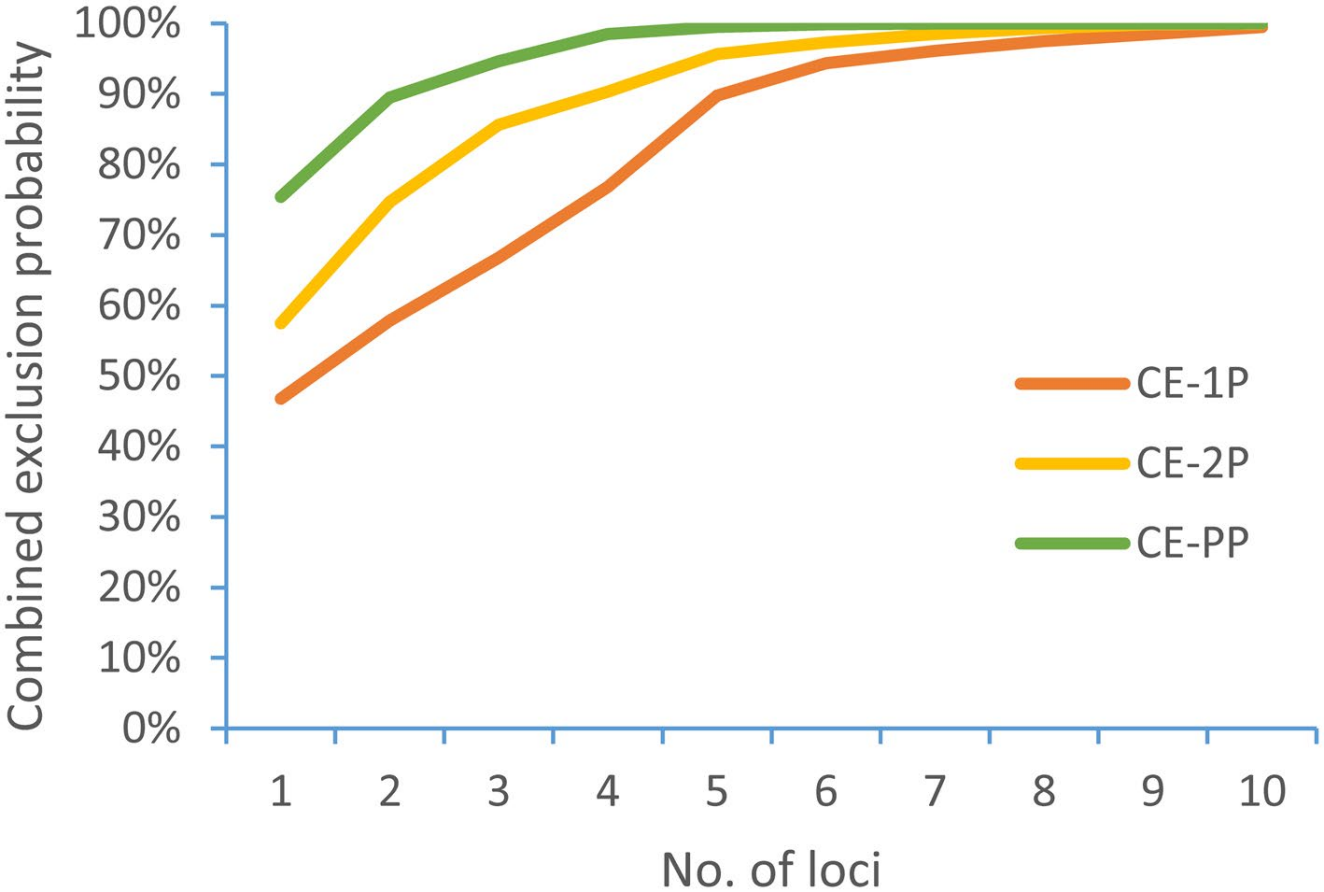
Combined exclusion probabilities based on the genotype of pair parent known (CE-PP), one parent known (CE-2P), and no parent known (CE-1P) of 10 microsatellites in a full-sib families of sterlet. Note: CE-1P: Combined exclusion probability (no parent known); CE-2P: Combined exclusion probability (one parent known); CE-PP: Combined exclusion probability (pair parent).

In this study, a total of 160 microsatellites primers were designed to develop duplex PCR reactions. 16 sterlet individuals were used to test the 160 microsatellites, and 10 microsatellites showed clearly polymorphism (Table 1). The observed allele number of the 10 microsatellites ranged from 2 to 6. The expected heterozygosity (*H*_*E*_), observed heterozygosity (*H*_*O*_), Shannon-Weiner diversity indices (*H′*) and polymorphic information content (*PIC*) of the 10 microsatellites ranged from 0.466 to 0.751, from 0.438 to 0.938, from 0.66 to 1.51 and from 0.368 to 0.716, respectively (Table 2). According to the results for the 10 microsatellites tested in the 16 sterlet individuals, we build five duplex PCR reactions (Table 1). The 16 sterlet individuals were accurately reconstructed in the dendrogram based in the 10 microsatellites by Mega software (Fig. 1). The five duplex PCR reactions were also used in the parentage analysis of the full-sib families of sterlet. Combined exclusion probability based on the genotype of pair parent known (CE-PP), one parent known (CE-2P), and no parent known (CE-1P) increased with the number of microsatellites (Fig. 2). Combined exclusion probabilities based on the genotype of CE-PP, CE-2P, and CE-1P of the 10 microsatellites were 99.99%, 99.96%, and 99.49%, respectively. Combined exclusion probability of CE-PP, CE-2P, and CE-1P can be reach 99% when used 10, 8, 5 microsatellites, respectively. These result showed that the 10 microsatellites should be helpful for parentage identification of sterlet.

**Table 1:** Characterization of five duplex PCR reactions in sterlet.

Groups	GenBank accession no	Primer sequences (5′ − 3′)	Annealing temperature (℃)	Repeat motif(s)	Length	Chromosomal Location
A	OM320532	F:GCGTTCACTGAGTCAATGCA	60	(TATC)_14_	222	28
		R:CTGGACAGAGAACAGATAGCGT				
	OM320533	F:ACCTGCCTTCTTCCAGCTTT		(AGAT)_12_	299	3
		R:AATCACGGACAGCCAAGAGG				
B	OM320534	F:ACAGTGGACAATGTGGCTCA	60	(TTTA)_10_	256	9
		R:CCAGGACCACGGCTAGTTTT				
	OM320535	F:TCACGTTGATCAGGGTCTTCA		(ATAG)_12_	181	9
		R:TCCACAAACACAAAACATTTGCT				
C	OM320536	F:ACCCTCCCACAGATGCTGTA	60	(TTTA)_8_	118	13
		R:TGATAGTTCAAATTGACGAAGGCT				
	OM320537	F:TCGGGGGTAAAATAATGGGAGA		(AATA)_9_	155	Unknow
		R:CTAACTCGGCCCCAAACCAT				
D	OM320538	F:GGCCACCACTGTTGATCTGA	60	(AAAT)_9_	229	16
		R:GCCATTCTCCTCCCTGACAC				
	OM320539	F:TGTGCTTACTTGCATCTGTTGT		(CTAT)_9_	303	23
		R:CGCTCCGCTCTAAGAAGACA				
E	OM320540	F:GGACATTCTCATTCTCAGCTGC	60	(ATAC)_9_	239	3
		R:ATCACGATCCATGCCTTGCA				
	OM320541	F:GCATGTGTCACTGAGATAGTTGC		(TATT)_9_	191	1
		R:TGCTGCAGTTGAGGTCCATT

**Table 2:** Genetic diversity of ten microsatellites in five duplex PCR reactions of sterlet. The table includes number of alleles observed (*Na*), observed heterozygosity (*H*_*O*_), expected heterozygosity (*H*_*E*_), Shannon–Wiener diversity indices (*H′*), polymorphic information content (*PIC*).

GenBank accession no	*Na*	*H*_*E*_	*H*_*O*_	*H'*	*PIC*
OM320532	4	0.717	0.750	1.32	0.680
OM320533	5	0.751	0.500	1.49	0.716
OM320534	4	0.533	0.750	0.96	0.515
OM320535	2	0.466	0.500	0.66	0.368
OM320536	4	0.686	0.688	1.26	0.653
OM320537	5	0.768	0.625	1.51	0.691
OM320538	4	0.658	0.813	1.22	0.642
OM320539	4	0.675	0.938	1.21	0.611
OM320540	4	0.492	0.438	0.95	0.539
OM320541	4	0.676	0.688	1.22	0.645

## Discussion

Application of microsatellite markers play important role in genetic conservation and management of fish, and many studies have demonstrated that microsatellite is usefulness for the broodstock management and assessment of population structures^22–26^. In the present study, five duplex PCR reactions were built to be used in the population genetics and parentage identification for sterlet. The microsatellite for sterlet have been developed in previous studies^12,27^. However, only seven tetranucleotide microsatellites were reported^12^. The risk of obtaining false alleles can be reduced by using tetranucleotide microsatellites instead of dinucleotide microsatellites^28^. The dinucleotide microsatellites are more likely to occur as shadow bands than tetranucleotide microsatellites during the PCR process^29^. Furthermore, tetranucleotide microsatellites are more stable and accurate than dinucleotide microsatellites^30^. Therefore, the 10 tetranucleotide microsatellites should be helpful for genetic study of sterlet.

The observed heterozygosity and expected heterozygosity of the 10 microsatellites in this study is higher than the previous microsatellites^12,27^. In this study, 9 of 10 microsatellites with *PIC* > 0.5, which is higher than the microsatellites that be reported in beluga sturgeon^31^, Amur sturgeon^10^, however, is lower than the microsatellites that be reported in Chinese sturgeon^6^, Dabry’s Sturgeon^9^. Although the method of alleles scoring (PAGE) used in this study is cheap and quick, it has many drawbacks, such as result interpretation error, misreading false allele and so on. In this study, there is no evidence of stutter bands, null alleles, or large allele dropout. The reason for this result may be due to the small sample size. These result suggested that the 10 microsatellites were helpful in studying of the population genetics of sterlet.

The previous study have showed that the CE-1P and CE-2P for parentage identification of Nile tilapia were 0.967 and 0.9999 by using 7 microsatellites^32^. Using four groups of fluorescent-labeled multiple capillary for parentage assignment, the assignment success rate of *O. niloticus*, *O. niloticus* × *O. aureus*, *O. aureus*, and their mixed population reached 100% when 7, 8, 9, and 12 microsatellites, respectively^33^. The parentage identification success rate was highly correlated with the allelic polymorphism of microsatellite^34^. The 10 microsatellites in this study have showed high allelic polymorphism, suggesting that they are suitable for parentage identification of sterlet. They can be useful tools to characterize the structure and genetic diversity of sterlet, to appropriate breeding schemes and establish broodstocks for supportive stocking programs, to aid the development of conservation measures as well as to monitor genetic changes in farmed strains. Although the 10 microsatellites presented here was meaning for sterlet parentage identification, this methodology still has some limitations: (a) The *PIC* values of the 10 microsatellites in this study are not sufficiently high to reduce the number of microsatellites for parentage assignment; (b) More stable microsatellites need to be further screened.

In conclusion, these five duplex PCR reactions, which consist of 10 microsatellites, should be helpful for assessing the population genetics and parentage identification of sterlet.

## Data Availability

Data are available from the corresponding author upon reasonable request.
